# Photoinduced bidirectional switching in lipid membranes containing azobenzene glycolipids

**DOI:** 10.1038/s41598-023-38336-x

**Published:** 2023-07-16

**Authors:** Jonas E. Warias, Franziska Reise, Svenja C. Hövelmann, Rajendra P. Giri, Michael Röhrl, Jule Kuhn, Malte Jacobsen, Kuntal Chatterjee, Thomas Arnold, Chen Shen, Sven Festersen, Andrea Sartori, Philipp Jordt, Olaf M. Magnussen, Thisbe K. Lindhorst, Bridget M. Murphy

**Affiliations:** 1 Institute of Experimental and Applied Physics, Kiel University, Leibnizstr. 19, 24118 Kiel, Germany; 2 Otto Diels Institute of Organic Chemistry, Kiel University, Otto-Hahn-Platz 3-4, 24118 Kiel, Germany; 3 Diamond Light Source, Harwell Science and Innovation Campus, Didcot, OX11 ODE UK; 4 Deutsches Elektronen-Synchrotron DESY, Notkestraße 85, 22607 Hamburg, Germany; 5 Ruprecht Haensel Laboratory, Kiel University, 24118 Kiel, Germany; 6 Present Address: Molecular Biophysics and Integrated Bioimaging Division, Lawrence Barkeley National Laboratory, 1 Cyclotron Road, Berkeley, CA 94720 USA; 7 Present Address: ISIS Neutron and Muon Source, Rutherford Appleton Laboratory, Didcot, OX11 0QX UK; 8 Present Address: Department of Chemistry, University of Bath, Claverton Down, Bath, BA2 7AY UK; 9 Present Address: European Spallation Source ERIC, P.O Box 176, 221 00 Lund, Sweden; 10 Present Address: ESRF-The European Synchrotron, 38043 Grenoble, France

**Keywords:** Biological physics, Surfaces, interfaces and thin films

## Abstract

Following the reaction of biological membranes to external stimuli reveals fundamental insights into cellular function. Here, self-assembled lipid monolayers act as model membranes containing photoswitchable azobenzene glycolipids for investigating structural response during isomerization by combining Langmuir isotherms with X-ray scattering. Controlled *in-situ trans/cis* photoswitching of the azobenzene N = N double bond alters the DPPC monolayer structure, causing reproducible changes in surface pressure and layer thickness, indicating monolayer reorientation. Interestingly, for monolayers containing azobenzene glycolipids, along with the expected DPPC phase transitions an additional discontinuity is observed. The associated reorintation represents a crossover point, with the surface pressure and layer thickness changing in opposite directions above and below. This is evidence that the azobenzene glycolipids themselves change orientation within the monolayer. Such behaviour suggests that azobenzene glycolipids can act as a bidirectional switch in DPPC monolayers providing a tool to investigate membrane structure–function relationships in depth.

## Introduction

Lipid membranes provide stability and a structural framework for biological cells. They ensure compartmentalization, protect and organize cells and maintain gradients. Understanding how the supramolecular chemistry and the biophysics of cell membranes are fine-tuned and controlled is an important topic^1^. In the past years, an increased interest in membrane dynamics and the interaction between membrane components has come into focus^2–4^ for supported bilayer membranes^5–8^ and vesicles^9^.

Introducing phtotoswitchable molecules within lipid membranes allows reversible structural changes to be introduced in a large area within the membrane without disturbing the membrane mechanics by external forces. This enables the possibility to study the membrane response to an internal conformational change^10^. Several published articles investigating photo switchable azobenzene-containing lipids confirm a switching response^12–14^ including the review by Beharry, A. A. and G. A. Woolley^11^. In the case of supported lipid bilayer membranes, a strong change in layer thickness upon reversible switching of azobenzene photolipids along with the possibility to tune the bilayer fluidity has been reported^5^.

However, the complexity of biological membranes makes direct investigation of biochemical and biophysical properties challenging. Lipid monolayers provide a simplified system for investigating model membranes at the liquid/air interface^15,16^. To learn about the effects of an external light stimuli on the monolayer behaviour, we have incorporated an azobenzene photoswitch in a Langmuir lipid monolayer^9,17,18^.

**Figure 1:**
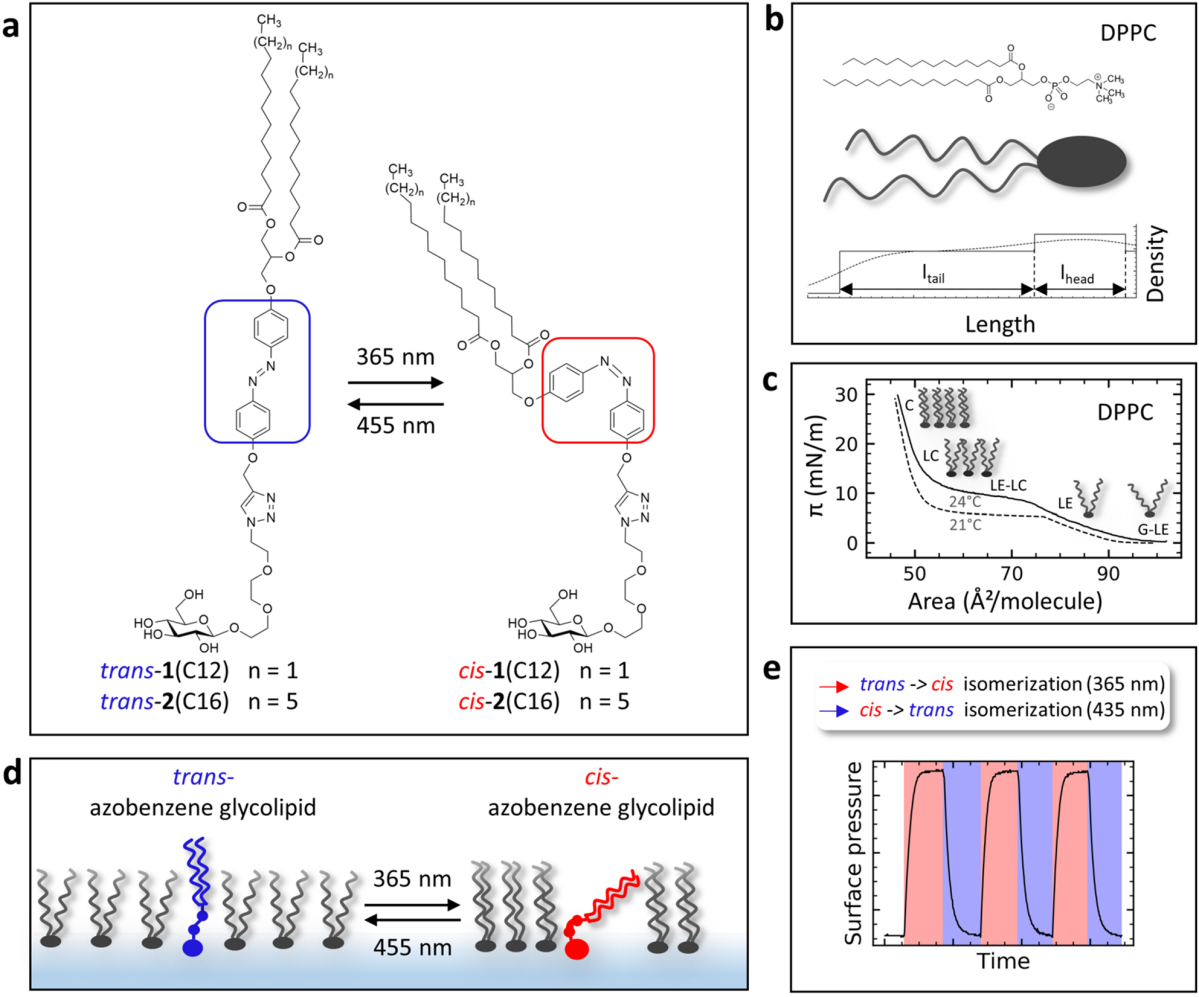
(**a**) Depiction of the trans- and cis-isomer, respectively, of the employed synthetic azobenzene glycolipids **1**(C12), and **2**(C16). They consist of a hydrophilic sugar head group, a central azobenzene photoswitch and two fatty acyl moieties of different chain lengths (12 and 16 carbon atoms, respectively). (**b**) Sketch of the DPPC (1,2-dipalmitoyl-phosphatidylcholine) molecule. The graph shows the scattering length density profile of a DPPC monolayer perpendicular to the surface, which can be obtained by X-ray reflectivity measurements, describing the length of the head (l_head_) and tail group (l_tail_) without (solid line) and with roughness (dotted line). (**c**) Pressure-area (π-A) isotherm of a DPPC Langmuir monolayer showing the typical gaseous/liquid expanded (G-LE), liquid-expanded (LE), liquid-expanded/liquid-condensed coexisting (LE-LC), liquid-condensed (LC) and condensed phase (C) under compression as described by Adamson^23^ at two temperatures 21 °C and 24 °C. (**d**) Model of monolayer compression upon photoswitching. e) The surface pressure response to photoswitching of azobenzene glycolipids embedded into a DPPC monolayer. The *trans*-azobenzene glycolipid derivatives can be converted to the *cis*-isomer by irradiation with UV light (365 nm) and the *trans*-state can be restored with visible light (455 nm) irradiation. Irradiation with 365 nm is depicted with red background and 455 nm with blue.

In this study, we employ 95/5% mixtures of DPPC (1,2-dipalmitoyl-phosphatidylcholine) and synthetic azobenzene glycolipids^19^. The azobenzene moiety serves as the photoswitch and can be isomerized between a straight *trans*-conformation and a bent *cis* form upon irradiation. *trans* → *cis* isomerization of the N = N double bond is induced with light of 365 nm, whereas back isomerization, *cis* → *trans*, occurs upon excitation with 455 nm (Fig. 1a, SI). It was possible to investigate the structural response of the membrane to photoswitching by an *in-situ* combination of Langmuir isotherms and X-ray scattering.

The investigation of Langmuir isotherms (Fig. 1c) is a well-established approach to study the structural phases^16^ of even challenging membrane structures^20^. In addition, X-ray scattering of lipid monolayers, provides information on membrane thickness, roughness and electron density in the direction perpendicular to the interface with nanoscale resolution (Fig. 1b)^21,22^.

The employed photoswitchable glycolipid mimetics consist of a hydrophilic β-d-glucosidic head group attached to a triethylene glycol linker and the azobenzene moiety. The hydrophobic tail group consists of a diacylglycerol esterified with two fatty acids containing either 12 or 16 carbon atoms, further referenced as **1**(C12) and **2**(C16) (Fig. 1a). In this account, we describe the properties of DPPC Langmuir isotherms containing 5% of **1**(C12) or **2**(C16), respectively, in response to photoisomerization of the embedded glycolipid photoswitch (Fig. 1d). In the following, we discuss changes of surface pressure behaviour (Fig. 1e). Furthermore, for the characterization of the photoisomeric lipid monolayers, we use X-ray reflectivity (XRR) to describe the observed structural rearrangement.

## Results and discussion

### Langmuir isotherms

**Figure 2:**
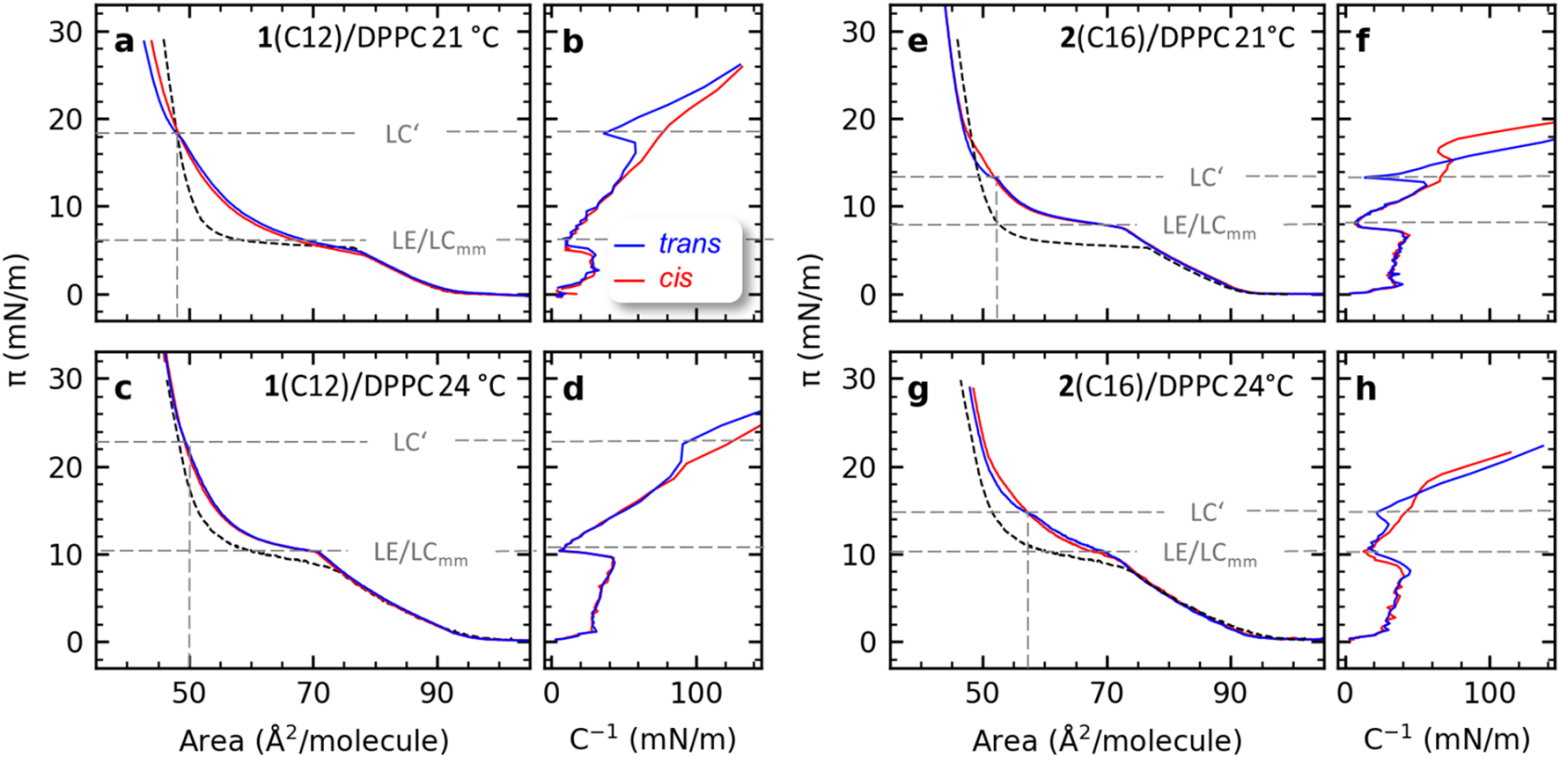
(**a**) The isotherms and (**b**) corresponding compression modulus C^-1^ plots for the mixed glycolipid **1**(C12)/DPPC monolayer for the *trans*- (blue) and the *cis*-state (red) and the pure DPPC (dotted line) at 21 °C. (**c**) Isotherms and (**d**) corresponding C^-1^ for the mixed glycolipid **1**(C12)/DPPC monolayer shown for the trans- (blue) and cis-state (red) at 24 °C. Horizontal dotted lines indicate the surface pressure region of the LE/LC phase transition for the mixed monolayers (LE/LC_mm_) for both trans and cis configurations, and for LC’ in the trans-state. The vertical dotted lines indicate the corresponding area per molecule (APM). (**e**, **g**) the isotherms for the mixed glycolipid **2**(C16)/DPPC and reference DPPC monolayers at 21 and 24 °C and (**f**, **h**) the C^-1^plots for the respective **2**(C16)/DPPC monolayers.

The effects induced by photoswitching of the glycolipid mimetic within the DPPC monolayers were analyzed. The isotherms of pure DPPC monolayers measured at 21 °C and 24 °C shown in Fig. 2 a, c, e and g were well in agreement with literature values and used as references for this study^24,25^. Following the isotherm description by Adamson^23^, we observe the typical gaseous/liquid-expanded coexisting (G-LE), liquid-expanded (LE), liquid-expanded/liquid-condensed coexisting (LE-LC), liquid-condensed (LC) and condensed phase (C) under compression (Fig. 1c). The well-known LE/LC phase transition^26^ is clearly visible for both investigated temperatures (21 °C and 24 °C) and shifts to lower area per molecule (APM) values and higher surface pressure with increasing temperature^25^.

For the fabrication of the photoswitchable Langmuir monolayers, DPPC was interlaced with 5% azobenzene glycolipid for **1**(C12) or **2**(C16), respectively. For both azobenzene glycolipid mimetics, stable monolayers at the air–water interface were formed, enabling reproducible compression isotherms up to 30 mN/m of surface pressure π (Fig. 2). APM values of the mixed glycolipid/DPPC monolayers were rounded to the values of a pure DPPC monolayer, neglecting the APM alteration caused by the different structures of the glycolipids.

### 1(C12)/DPPC

For the **1**(C12)/DPPC monolayer at 21 °C and 24 °C, the expected LE/LC phase transition for the mixed monolayer (LE/LC_mm_) is clearly visible and exhibits behaviour similar to the pure DPPC transition marking the rearrangement of DPPC in the monolayer as seen in Fig. 2a, and c . LE/LC_mm_ shows a shift to lower area per molecule (APM) values and higher surface pressure with increasing temperature, a behaviour also seen in DPPC. However, in the mixed monolayers in both isomeric states, *trans* and *cis* the LE/LC_mm_ is broader and is shifted to slightly higher surface pressure than LE/LC for pure DPPC at 21 and 24 °C. Also, the *trans*-**1**(C12)/DPPC monolayer isotherm clearly displays a further discontinuity which we name LC’, close to 18 and 22 mN/m, at 21 and 24 °C respectively. LC’ indicates a rearrangement of the photoswitch within the *trans*-monolayer into a second orientation of the *trans*-conformation. In the compression modulus $C^{ - 1}$ plots, where $C^{ - 1} = - Ad\pi /dA$ (Fig. 2b/d), the dip representing LC’ becomes less pronounced with increasing temperature, while the LE/LC phase transition dip remains constant. Further, no clear indication of a conformation change is observed for the *cis*-**1**(C12)/DPPC monolayer. Comparison of the *trans*- and *cis*-**1**(C12)/DPPC isotherms in the regime between the LE-LC coexistence phase and LC’ shows the surface pressures *π* are slightly higher for the *trans*-isomer than for the *cis*-isomer. Interestingly, the behaviour is reversed at APMs below LC’. Hence, LC’ appears to be a crossover point, indicating a deviating structural orientation within the monolayer between both photoisomers.

### 2(C16)/DPPC

The isotherms for the *trans*- and *cis*-**2**(C16)/DPPC monolayers at 21 °C and 24 °C show similar behaviour to the analogous mixed monolayers containing **1**(C12). The same slight shift of LE/LC_mm_ to lower APM and higher surface pressure values occurs with increasing temperature. Again a reorientation LC’ is seen here at close to 14 and 15 mN/m for 21 and 24 °C respectively (Fig. 2e–h) and again becoming less pronounced with increasing temperature. For both samples a shift to higher surface pressures with increasing temperature is observed, though, the shift to higher surface pressures is much smaller for the *trans*-**2**(C16)/DPPC sample. The observed temperature dependence of the isotherm is expected and in accordance with previously described effects on DPPC^27^.

### Comparison

Comparing isotherms at the same temperatures for both *trans*-**2**(C16)/DPPC and *trans*-**1**(C12)/DPPC, LC’ occurs at lower surface pressures for *trans*-**2**(C16)/DPPC. Again, a crossover point as seen for **1**(C12)/DPPC is observed, endorsing a deviating structural orientation within the monolayer between both photoisomers. In contrast to the *cis*-**1**(C12)/DPPC monolayers data, a clear LC’ reorientation is observed in the *cis*-**2**(C16)/DPPC isotherms at 21 °C (Fig. 2e, f) and may also occur at 24 °C (Fig. 2g, h). LC’ moves to higher surface pressure with increasing temperature for both C12 and C16 but the relative difference in surface pressure between LE/LC_mm_ and LC’ is very strongly related to the change in chain length of the fatty acyl tail groups from 12 to 16 carbon atoms. Indeed, LC’ for *trans*-**2**(C16)/DPPC occurs at higher APM and higher surface pressure than for *trans*-**1**(C12)/DPPC. The Langmuir isotherm measurements show the same qualitative behaviour for both mixed monolayer samples at **1**(C12)/DPPC and **2**(C16)/DPPC at 21 °C and 24 °C.

### Reversible photoswitching of Langmuir isotherms at constant APMs

**Figure 3:**
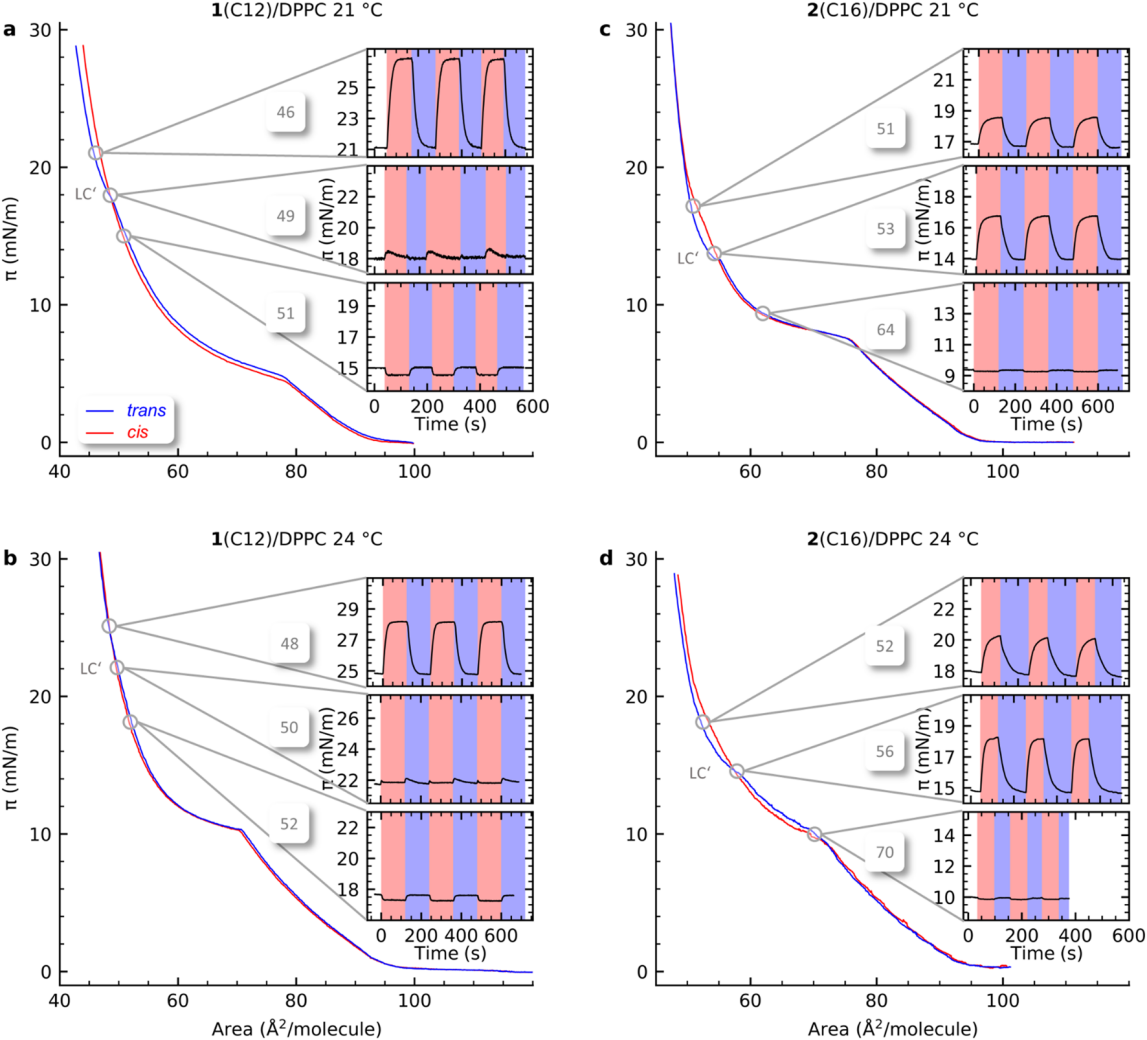
Isotherm for the mixed glycolipid **1**(C12)/DPPC monolayer are shown in (**a**, **b**) and for **2**(C16)/DPP monolayer in (**c**, **d**) at 21 °C and 24 °C for *cis*- (red) and *trans*-conformation (blue). Insets show the surface pressure response to photoswitching of embedded azobenzene glycolipids at selected APMs below, close to and above LC’. The switching from *trans* to *cis* and back was induced by irradiation with UV (365 nm) depicted with red background and visible light (455 nm), depicted with blue background. Data are shown on the same scaling for ease of comparison. Switching was always started from the *trans* isotherm. The insets are further detailed sin the supporting information (Figure S2).

In the next step, photoswitching response experiments were carried out at three constant APM values for each mixed monolayer (**1**(C12)/DPPC and **2**(C16)/DPPC) at both 21 and 24 °C. The APM values were chosen in regard to their relative position to LC’ of the respective *trans*-isotherms and will be referred to as above, below and close to LC’. Repeated irradiation of the Langmuir films was carried out with UV light of 365 nm to effect *trans* → *cis* isomerization of the azobenzene glycolipid and visible light of 455 nm to effect *cis* → *trans* isomerization. A reproducible cyclic change between the *trans-* and the *cis*-configuration was observed (Fig. 3). The data shown are always started from the *trans*-isotherm. Starting from the *cis*-isotherm leads to the same results. The comparison is shown in the supporting information.

The pressure changes (Δ*π*) measured upon photoswitching are reversed above and below LC’ as listed in Table 1. At an APM value above LC’, *π* decreases by 2–5% for **1**(C12)/DPPC and 1% for **2**(C16)/DPPC upon *trans* → *cis* switching at both temperatures. At APM below LC’, the same switching process results in a large increase in *π* of 13 and 26% for **1**(C12)/DPPC and 9 and 13% for **2**(C16)/DPPC at 21 and 24 °C respectively. This is clear evidence that the chain length of the embedded azobenzene glycolipids **1** and **2** influences the pressure change below and above LC’ upon photoisomerization. Our findings differ from previous studies with lipid monolayers containing azobenzene derivatives, which report an increase of Δ*π* over the entire isotherm upon *trans* → *cis* switching^17,28^.

**Table 1:** Measured parameters of **1**(C12)/DPPC and **2**(C16)/DPPC monolayers upon photoswitching: surface pressure change (Δ*π*_*trans→cis*_), membrane switching times (*τ*) and changes in total layer thickness (Δ*l*_*trans→cis*_).

Area (Å^2^/molecule)	*π*_*trans*_^a^ (mN/m)	Δ*π*_*trans→cis*_ (mN/m)	*τ*_*cis→trans*_ (s)	*τ*_*trans→cis*_ (s)	Δ*l*_*trans→cis*_ (Å)
**1**(C12)/DPPC 21 °C					
51 (> LC’)	15	− 0.7 ± 0.05	6.4 ± 0.3	3.6 ± 0.8	− 0.7 ± 0.7
49 (≈ LC’)	18	0.0 ± 0.05	2.0 ± 1.0^b^	3.7 ± 0.5^b^	0.0 ± 0.2
			3.5 ± 0.5^c^	42.0 ± 5.0^c^	
46 (< LC’)	21	5.5 ± 0.2	19.7 ± 0.8	14.8 ± 0.9	0.2 ± 0.2
**1**(C12)/DPPC 24 °C					
52 (> LC’)	18	− 0.4 ± 0.1	5.3 ± 0.5	5.6 ± 0.5	
50 (≈ LC’)	22	0.0 ± 0.2	3.8 ± 0.2	1.0 ± 1.0	
			52.0 ± 5.0	4.3 ± 0.2	
48 (< LC’)	25	3.4 ± 0.2	16.4 ± 0.2	12.8 ± 1.0	
**2**(C16)/DPPC 21 °C					
64 (> LC’)	9.5	− 0.1 ± 0.02	6.3 ± 1.0	4.1 ± 1.0	
53 (≈ LC’)	14	2.7 ± 0.1	25.0 ± 3.0	15.8 ± 0.2	
51 (< LC’)	17	1.6 ± 0.1	23.3 ± 1.0	20.0 ± 0.4	
**2**(C16)/DPPC 24 °C					
70 (> LC’)	10	− 0.1 ± 0.02	6.9 ± 1.2	4.5 ± 1.3	0.0 ± 1.2
56 (≈ LC’)	15	3.4 ± 0.1	29.0 ± 0.9	12.8 ± 0.2	3.3 ± 1.0
52 (< LC’)	18	2.3 ± 0.1	30.4 ± 0.6	19.1 ± 0.8	1.5 ± 1.0

^a^*π*_*trans*_ indicates the surface pressure of the *trans*-isotherm at the respective APM values before photoswitching.

^b^Time for the increase during transient pressure behaviour.

^c^Time for the decay during transient pressure behaviour.

At APM values close to LC’ (49 Å^2^/molecule), the long-term steady state pressure change for the *trans* → *cis* switching converges to Δ*π* ≈ 0 mN/m for the **1**(C12)/DPPC isotherm at both temperatures (Fig. 3a, b). One explanation for the disappearance of the observed pressure change is a diverging orientation of the conformers coexisting within the monolayer at the crossover point. One conformation causing an increase of pressure and the other one compensating for it. For the **2**(C16)/DPPC membrane such a point could not be observed directly at the crossover point, maybe due to the stronger relaxation of the membrane. The measurement obtained closest to LC’ displayed the behaviour similar to the measurement below LC’ resulting in an increase by 19–23% for the **2**(C16)/DPPC membrane close to LC’.

The switching reveals clear similarities between the two samples and varies little with the increase of temperature from 21 to 24 °C. A relatively small decrease up to 5% in surface pressure was observed at APM values above LC’. At APM’s below LC’ a much larger increase up to 26% was observed.

### Membrane switching kinetics

The isomer-dependent phase transition LC’, the difference in Langmuir isotherms between **1**(C12)- and **2**(C16)-mixed monolayers as well as the pressure responses above and below LC’ indicate complex structural changes within the azobenzene glycolipid/DPPC monolayers. In order to further investigate the structural response caused by the photoswitching the mixed glycolipid/DPPC monolayers, the kinetics of the photoswitching process were determined, in particular the membrane switching time *τ*.

The membrane switching times for the *trans* → *cis* (*τ*_*trans→cis*_) and the reverse isomerization (*τ*_*cis→trans*_) were calculated from the average surface pressure changes for each APM value (Fig. 3, SI for experimental details) and are listed in Table 1. For both monolayers, *τ* increases at lower APM values indicating lower switching efficiencies with rising surface pressure. For both monolayers at APM values above and below LC’, the membrane switching times for the *cis* → *trans* isomerization are mostly longer than for the reverse switching i.e. *τ*_*trans→cis*_ is faster than *τ*_*cis→trans*_. Although *π* decreases for APMs above LC’ and increases for those below upon irradiation_,_ we note that the membrane relative switching time difference between *τ*_*trans→cis*_ and *τ*_*cis→trans*_ does not. The switching to higher surface pressures is intrinsically unfavourable, as the resulting membrane switching times are predominantly determined by the membrane density and the excitation efficiency of the photoswitch. Additionally, for **1**(C12)/DPPC the membrane switching time *τ*_*trans→cis*_ observed at the crossover point within LC’ displays a transient pressure behaviour upon irradiation (Fig. 3a, b). This effect was only observed at the crossover point, indicating that a rearrangement of the monolayer precedes conformational equilibrium. The initial increase of the surface pressure and the following decrease can be described by an exponential function (Table 1). In contrast to the behaviour far above and below LC’, at the crossover we observe that the overall membrane switching displays a more varying behaviour. At 21 °C *τ*_*trans→cis*_ is slower than *τ*_*cis→trans*_, by a factor of 2 for the pressure increase and by one order of magnitude for the decay. At 24 °C the opposite behaviour is observed (*τ*_*trans→cis*_ is faster than *τ*_*cis→trans*_). We were not able to tune the temporal behaviour around LC’ by slight variation of the APM.

### X-ray reflectivity

X-ray reflectivity** (**XRR) provides an orthogonal type of measurement granting access to further insights regarding the structural changes observed upon irradiation within the photoswitchable monolayers. XRR data were collected *in-situ* during irradiation of the monolayers at APM values identical to the corresponding compression isotherm measurements. The measured specular reflectivity intensities $R$ were normalized by the Fresnel intensity for a perfect optically flat surface *R*_*F*_ and were used to derive the scattering length density (*SLD*) of the monolayers. The *SLD* values were fitted using a two-slab model distinguishing the tail (*l*_*tail*_) and head (*l*_*head*_) of the membrane molecules with *l*_*total*_ describing the total length. Details of the fitting process are given in the experimental section with fitting parameters listed in the supporting information (Table S2). The measurements for **1**(C12)/DPPC and **2**(C16)/DPPC where taken at two different temperatures 21 and 24 °C. The isotherm data shown above account for the different measurement temperatures and allow evaluate the measured data in context of the temperature offset.

**Figure 4:**
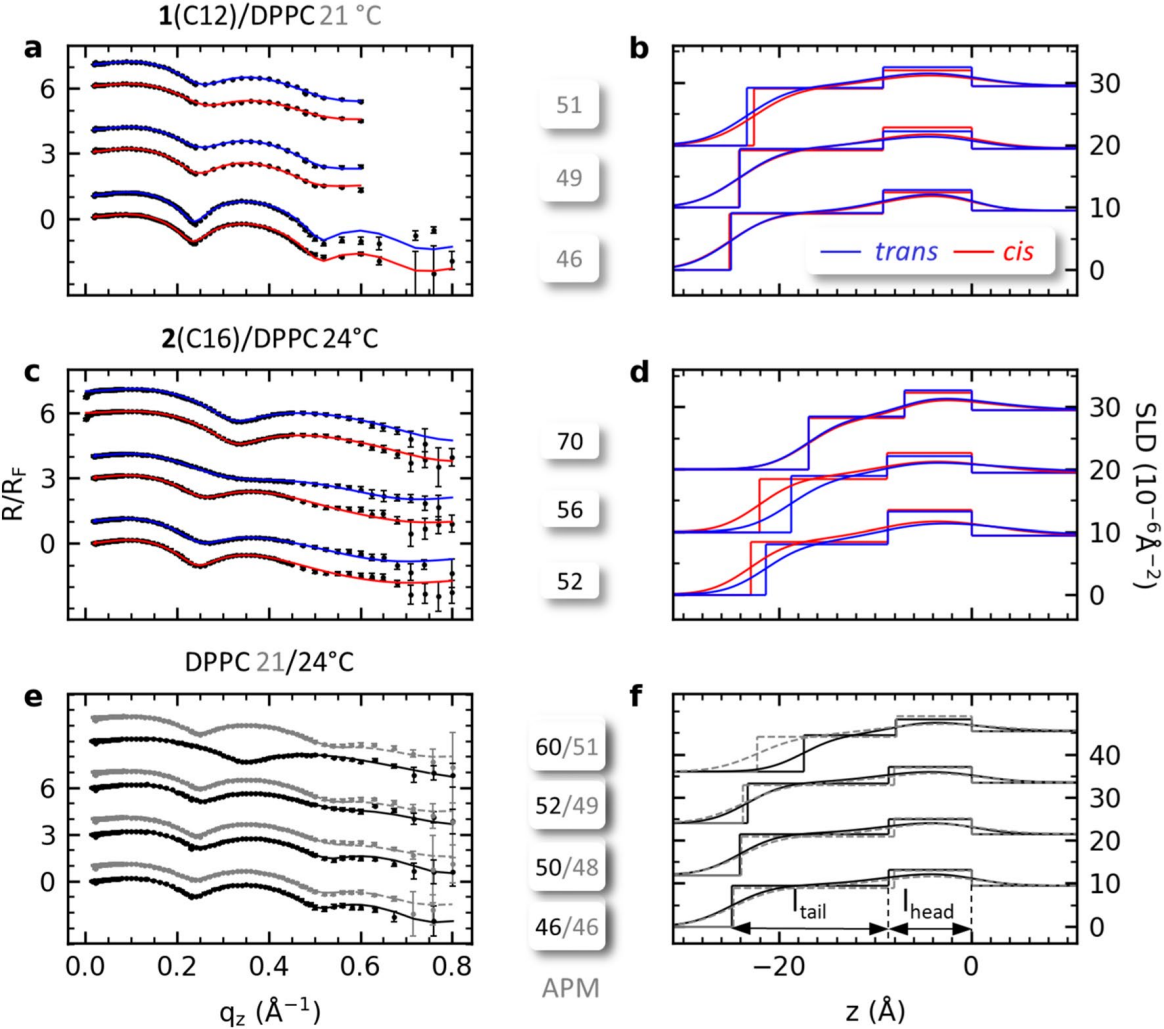
Fresnel normalized XRR data with the model fit (left) and the corresponding scattering length density *SLD* (right) for **1**(C12)/DPPC (**a**, **b**) at 21 °C and **2**(C16)/DPPC (**c**, **d**) at 24 °C. The *trans*-monolayer is marked in blue while the *cis*-monolayer is depicted in red. DPPC monolayer at 21 °C (dashed grey line) and 24 °C (solid black line) are shown in (**e**, **f**). The measurements were made at APMs above, close to and below LC’ at identical APM values to the switching experiments (cf. Fig. 3). The data are vertically offset for clarity.

In all XRR curves, pronounced oscillations (Kiessig fringes) are observed (Fig. 4a,c,e), which indicate a good integrity of lipid monolayers on the water surface. The fringe minima shift towards lower momentum transfer vector (*q*_*z*_) values for the membranes measured at lower APM values. This indicates an increase in layer thickness under compression.

Pure DPPC monolayers were measured at 21 and 24 °C as reference (Fig. 4e,f). Compressing the DPPC membrane from an expanded state at 10 mN/m to a more compressed state at 30 mN/m leads to an increase in total molecular length *l*_*total*_ from 22.3 Å to 24.7 Å at 21 °C and from 17.5 Å to 25.0 Å at 24 °C. With decreasing APM, the molecules condense into a tighter packing, leading to an elongation of the molecules and an increase in the scattering length density (*SLD*). Despite the strong increase in pressure, compressions below an APM value of 49 Å^2^/molecule only lead to a marginal increase in *l*_*total*_ of the DPPC molecules, suggesting an approach to the tightest packing possible for the molecular structure. Also, the difference in temperature has no measured influence on *l*_*total*_ for three of the measured surface pressures at 15, 18 and 30 mN/m. *l*_*total*_ is identical within the error bars. Only at 10 mN/m a larger difference was observed, which is due to the higher order of the DPPC molecules at lower temperatures. Hence, the results are in good agreement with theory^23^ and previously reported studies^29,30^.

**Figure 5:**
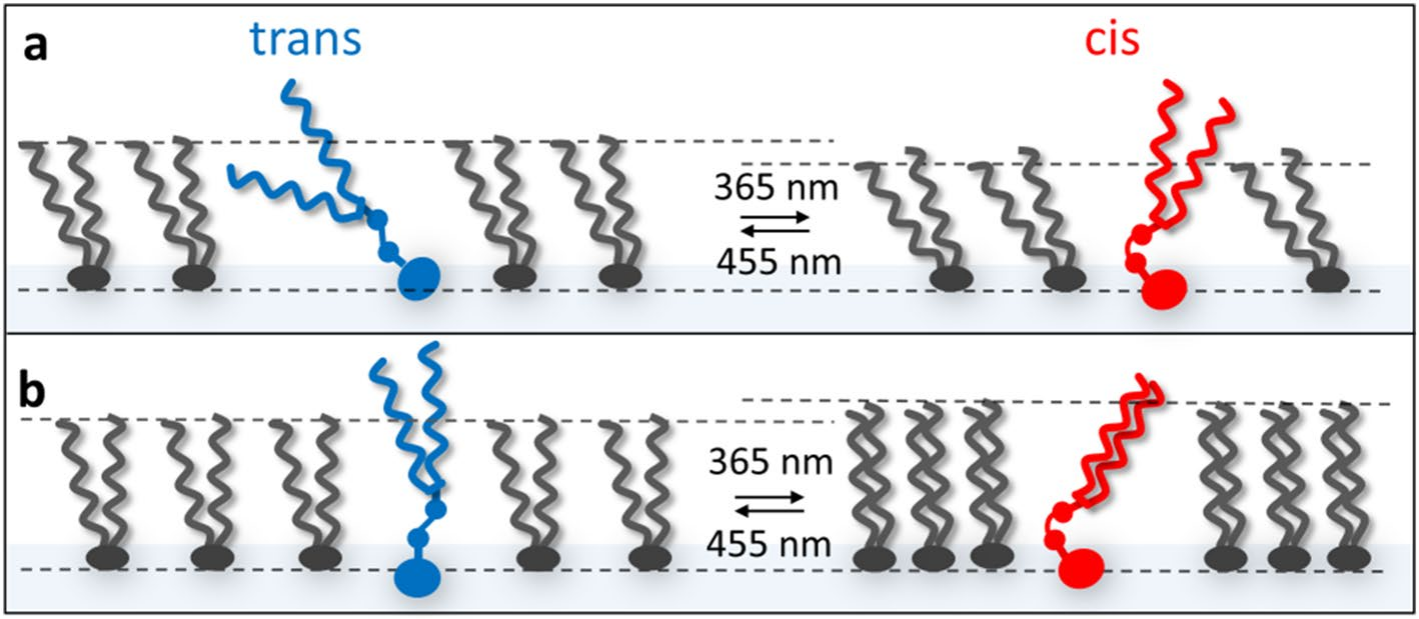
Monolayer relaxation and compression upon photoswitching at area per molecules a) above and b) below LC’ for the *trans* (blue) and *cis* (red) state upon visible (455 nm) and UV (365 nm) illumination. We propose that compression of the monolayer induces a conformation change of the embedded photoswitch.

The glycolipid/DPPC mixtures, both with embedded glycolipid **1**(C12) (Fig. 4a, b) and **2**(C16) (Fig. 4c, d), show the same trend of increasing monolayer thickness *l*_*total*_ upon compression as the pure DPPC monolayer. Upon *trans* → *cis* photoswitching at APMs above LC’, the average total length *l*_*total*_ of the **1**(C12)/DPPC monolayer decreases by 0.6 Å. In conjunction with the pressure change of Δ*π* = -− 0.7 mN/m measured at the corresponding APM of 51 Å^2^/molecule, the decrease in membrane thickness confirms a relaxation of the **1**(C12)/DPPC monolayer upon isomerization to the *cis*-state. This allows for the monolayer molecules to readjust towards less aligned and less upright orientations (Fig. 5b). On the other hand, a decrease in membrane order upon *trans* → *cis* photoswitching indirectly indicates a reduction of space occupied by the photoswitchable glycolipid mimetic, either in the form of a more condensed molecule or a less demanding interaction with its surroundings. In contrast, for the measurements at APMs close to (49 Å^2^/molecule) and below LC’ (46 Å^2^/molecule) no significant change of the thickness of the **1**(C12)/DPPC monolayer was observed upon irradiation, despite an increase in surface pressure of Δ*π* = 5.5 mN/m. In accordance with the observations for the reference DPPC monolayer, the minor increase in molecular length (*l*_*total*_) is based on the tight packing of the membrane molecules at lower APMs resulting in proportionately higher changes in *π* upon structural perturbation^30^.

*Trans* → *cis* photoswitching of the **1**(C12)/DPPC membrane in the pressure regime close to LC’ resulted in a decrease of the tail density (*SLD*_*tail*_) from 9.4 × 10^-6^ Å^-2^ to 9.2 × 10^-6^ Å^-2^, whereas the measured density of the head group (*SLD*_*head*_) increased from 12.2 × 10^-6^ Å^-2^ to 12.9 × 10^-6^ Å^-2^ (Fig. 4b). Even though an influence of the fitting process on the resulting values for *SLD*_*tail*_ and *SLD*_*head*_ cannot be excluded, this shift in electron density values cannot be explained by the applied fit. In consequence, the reallocation of *SLD* from the hydrophobic part of the membrane towards the head group can be attributed to a condensation of electron density in the fitted head group of the *cis*-conformer of **1**(C12) in comparison to the *trans*-configured counterpart. This reallocation of *SLD* into the head group is due to the switching-induced reorientation within the monolayer at the crossover point leading to the transient pressure increase observed. This could also play a part in causing the longer membrane switching time.

The **2**(C16)/DPPC monolayer displays no changes in *l*_*total*_ upon photoswitching at APMs above LC’. At APMs close to LC’ (56 Å^2^/molecule), photoswitching induced an increase in the total monolayer length *l*_*total*_ by 17% (Δ*l*_*trans→cis*_ = 3.3 Å) to 22.1 Å. In conjunction with the pressure increase of Δ*π* = 3.4 mN/m, the increase in the total length provides further indication of a higher ordering in the monolayer upon photoswitching (Fig. 5a). Compressing the membrane to APM values significantly below LC’ (52 Å^2^/molecule) leads to a reduction of photoswitching-induced structural change, increasing *l*_*total*_ by only 1.5 Å or 7%, compared to the measurement closer to LC’ (56 Å^2^/molecule). The compression also leads to a suppression of the change in surface pressure (Table 1). This decline is attributed to steric hindrance of the tightly packed molecules preventing isomerization. A similar inhibition of photoswitching in compact azobenzene-containing Langmuir films has been reported previously by Liu and coworker^28^. Comparable to the **1**(C12)/DPPC sample *SLD*_*tail*_ decreases from 9.0 10^-6^ Å^-2^ to 8.5 10^-6^ Å^-2^ upon isomerization for the **2**(C16)/DPPC at the measurement closest to LC’, whereas the *SLD*_*head*_ increases from 12.2 10^-6^ Å^-2^ to 12.7 10^-6^ Å^-2^ (Fig. 4d), likewise indicating a shift within the membrane towards a more condensed head group alignment upon switching.

The **2**(C16)/DPPC membrane displays a contrasting behaviour to the **1**(C12)/DPPC membrane upon isomerization with respect to the total molecular length *l*_*total*_ (Fig. 4b). The divergent behaviour between the membranes embedded with either **1**(C12) or **2**(C16) can be traced back to the pressure regimes at which LC’ is observed. In the case of the glycolipid **1**(C12)/DPPC membrane LC’ is located in a surface pressure domain comparable to the C region of a monolayer consisting only of DPPC. Therefore, the monolayer displays only a slight change in *l*_*total*_ (Δ*l*_*trans→cis*_ = 0.2 Å) at APMs below LC’, despite being coupled with a pressure increase of Δ*π* = 5.5 mN/m, due to the highly ordered membrane. For **2**(C16)/DPPC on the other hand, LC’ is observed at higher APMs comparable to the less dense ordered LE-LC/LC phase region, leading to a strong response of the membrane close to and below LC’ upon photoswitching. At APMs above LC’ almost no impact upon photoswitching in the unordered membrane (Δ*l*_*trans→cis*_ = 0.0 Å, Δ*π*_*trans→cis*_ = -0.1 mN/m) is observed because any reorientation of the photoswitchable glycolipid is compensated by the loose conformation of the membrane. The switching behaviour of both monolayers at pressures resembling the LE-LC/LC regime of DPPC monolayers show a considerable change in *l*_*total*_ upon photoswitching. Despite the illustration in Fig. 5 a phase separation between the azobenzene glycolipid and the DPPC in connection with H-aggregation as highlighted in the literature^5^, could be possible but was neither observed within the X-ray data or our Brewster angle microscope with a resolution of 12 µm.

**Figure 6:**
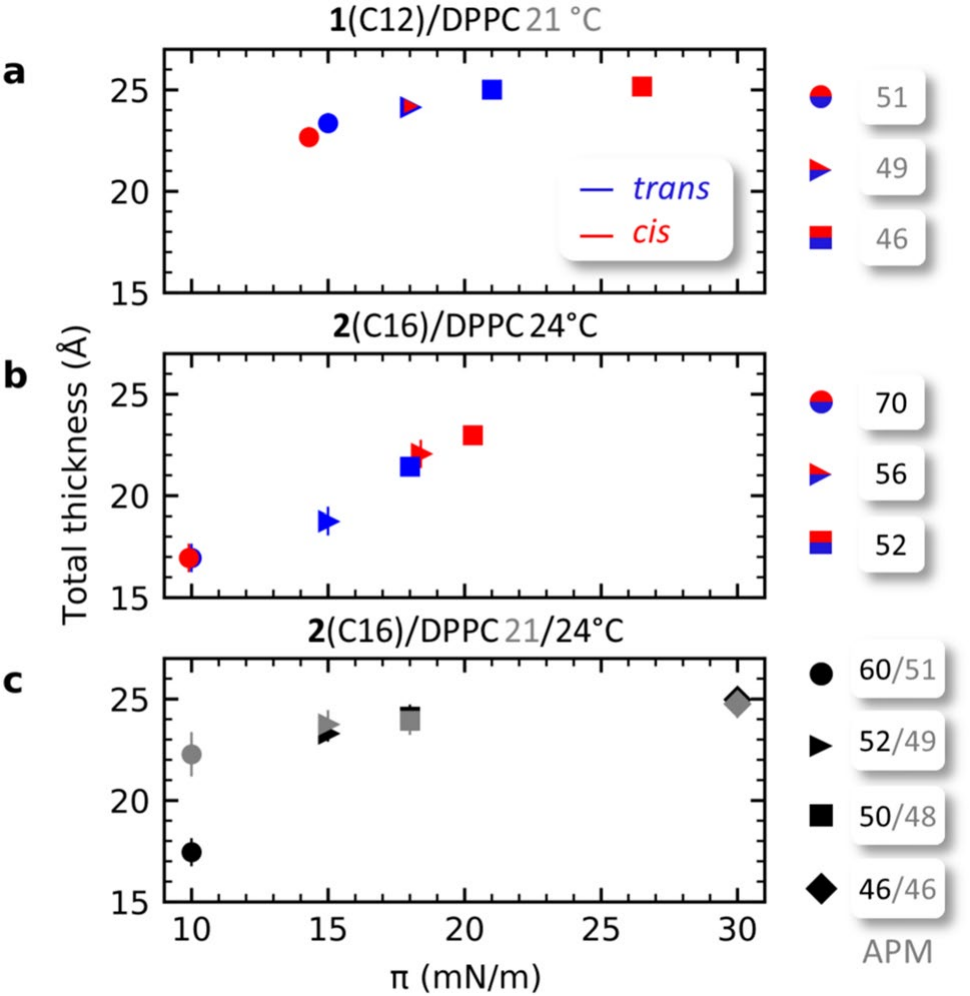
Change of total thickness over surface pressure of monolayers in the *trans-* (blue) and *cis*-state (red) for **1**(C12)/DPPC (**a**) and **2**(C16)/DPPC (**b**) mixtures, as well for pure DPPC at 21 and 24 °C (**c**). The measurements were made at APMs above, close to and below LC’ at identical APM values as in the switching experiments (cf. Fig. 3).

In order to give a more coherent overview of the structural changes induced by photoswitching throughout the different pressure regimes, the values for *l*_*total*_ determined by XRR were plotted against the surface pressure change (Fig. 6). The observed switching behaviour underlines the importance of the LE-LC/LC phase for a facile structural switching response.

At all APM values measured for the** 2**(C16)/DPPC XRR experiments, a significant reduction of the head-water roughness σ_water_ was observed following *trans* → *cis* photoisomerization. The decrease in σ_water_ corresponds to a more uniform alignment of the membrane head groups at the water interface. As an azobenzene glycolipid **2**(C16) molecule is longer than a DPPC molecule, this indicates that the head group of the azobenzene glycolipid is more aligned to the DPPC head group in the *cis*- than in the *trans*-state.

With the XRR we see reproducible changes within the lateral membrane structure upon photoswitching in the region of the LE/LC phase transition. Within the LE and C phase no changes in the lateral structure where observed. In future grazing incidence X-ray diffraction would be a welcome addition to the techniques for determining the precise structure.

## Conclusion

Stable self-assembled DPPC Langmuir monolayers with 5% embedded glycolipid azobenzene photoswitches with a tail chain length of 12 or 16 carbon atoms, respectively, were formed at an air–water interface. Irradiation with light stimulated perturbation within the hybrid monolayers due to reversible *trans*/*cis* isomerization of the N = N azobenzene double bond. The monolayer switching response was evaluated by a combination of Langmuir isotherm and X-ray reflectivity measurements. We observe a conformation change (LC’) within the Langmuir isotherms measured for the *trans*-state of all prepared membranes. LC’ is caused by rearrangements of the molecules within the membrane induced by reorientation of the embedded photoswitchable glycolipid in response to a decrease in APM. Within the cis-state a clear LC’ conformation change was only observed for the 2(C16)/DPPC monolayer at 24 °C. While in previous studies of azobenzene-containing mixed monolayers a broadening of the LE/LC phase transition was reported, such a distinct additional discontinuity similar to LC’ was not observed^17,28^. The reorientation of the photoswitch within the membrane leads to a reversal of photoswitching membrane response above and below LC’ with the cross over occurring at LC’. At APMs below LC’, the area occupied by the azobenzene glycolipids increases upon *trans* → *cis* isomerization (after irradiation with UV light), resulting in elevated surface pressures *π*. In contrast, at APMs above LC’ the opposite behaviour is observed. Here, a decline in *π* indicates a reduction of the area occupied by the azobenzene glycolipids in the *cis*-state compared to the *trans*-state indicating that the embedded mimetics **1**(C12) and **2**(C16) rearrange to a more upright position at LC’.

From the presented data, the following assumptions can be derived: At APMs above LC’ the membrane including the *trans*-configured photoswitch is in a less ordered configuration leading to more tilted orientations of the DPPC molecules (Fig. 5a). In consequence, the area occupied by the azobenzene photoswitch in the more twisted *cis*-conformer is decreased alongside the overall length of the glycolipid, leading to a decrease in surface pressure. The overall decrease in total layer thickness *l*_*total*_ upon photoswitching at APMs above LC’ supports this claim as the increase in available area is followed by the relaxation of the DPPC matrix for the **1**(C12)/DPPC monolayer leading to a reduction in monolayer thickness. In the case of **2**(C16)/DPPC only a negligible decrease in *l*_*total*_ was observed for APM values above LC’. Due to the occurrence of LC’ at higher APM values for the membrane containing **2**(C16) any reorientation of the photoswitch is compensated by the loosely structured membrane as it is close to the LE phase. Approaching APMs close to and below LC’ a more upright and aligned conformation is forced upon the photoswitchable glycolipid in the *trans*-state leading to the observed second phase transition following compression. At the crossover point, the ongoing change in configurations alleviates the increase in pressure exerted upon the membrane leading to mitigation of the surface pressure increase upon irradiation. For APMs below LC’, the **2**(C16)/DPPC membrane displays an increase of surface pressure and layer thickness, while for the **1**(C12)/DPPC monolayer, only a surface pressure increase was observed upon photoswitching to the *cis*-state. The structural changes observed upon photoswitching are strongest in the LE-LC/LC regions, which occurs for the **1**(C12)/DPPC monolayer above LC’ and close to LC’ for the **2**(C16)/DPPC monolayer.

The shift of the LC’ region to higher APMs for the **2**(C16)/DPPC membrane compared to the monolayer containing the shorter **1**(C12) indicates a dependency on the chain length of the photoswitch providing possibilities to fine-tune the system. The capability to adjust not only the operational regime of the photoswitch, but furthermore fine-tune the induced structural response upon isomerization, opens up approaches for membrane research and applications. Incorporation of photoswitchable lipid mimetics in DPPC monolayers is a promising step towards the formation of photosensitive vesicles for future medical applications such as drug delivery^12,14^. Further investigation of membranes with embedded photoswitchable azobenzene glycolipids provide a viable pathway to understand the kinetic behaviour of proteins within membranes^31^ and molecular recognition processes at the cell surface such as carbohydrate-lectin interactions^32^.

## Experimental

### Materials

1,2-dipalmitoyl-phosphatidylcholine (DPPC) was purchased from Avanti Polar lipids (Alabaster, AL). The azobenzene glycolipid mimetics **1**(C12) and **2**(C16) were synthesized as described in accordance with our previously published synthesis route^19^. The lipids were dissolved in chloroform (Aldrich) with a concentration of 1 mM and mixed in a concentration ratio of 5:95 azobenzene glycolipid/DPPC for the compression isotherm and XRR studies.

### Langmuir compression isotherms

Following a thorough cleaning with ethanol and three times Milli-Q water flushing, lipid solutions and mixtures were spread on the Milli-Q water surface in an R&K Langmuir Trough (Potsdam, Germany) with a gas-tight microsyringe (Hamilton). The trough has a total area of 502 cm^2^ with one moving barrier and was used for experiments at DESY and our institute laboratory. Isotherms were measured by a film balance with a 3 mm wide filter paper as Wilhelmy plate. All isotherm experiments were carried out with a compression rate of 14 cm^2^/min. Measurements at the Diamond science facility were conducted in a Nima Langmuir trough with one barrier and a total area of 800 cm^2^ (**1**(C12) X-ray measurements).

### Isomerization

The illumination device consisting of a row of three 365 nm LEDs (Nichia, NCSU033B(T)) and three 455 nm LEDs (Osram, LD CQ7P) was mounted on top of the trough outside a UV transparent glass window to switch between the two configurations of the azobenzene glycolipids. The measured fluencies at the monolayer position were 1.1 mW/cm^2^ for the 365 nm and 1.1 mW/cm^2^ for the 455 nm wavelength. For the X-ray measurements at Diamond Light Source the LED row was mounted inside the existing enclosure with a fluency of the visible wavelength of 1.2 mW/cm^2^.

### Kinetic switching experiments

The Langmuir switching experiments were carried out at the R&K Langmuir Trough. First an azobenzene glycolipid/DPPC monolayer in *trans*-state was prepared and compressed to the designated APM values. After at least 15 min waiting time the layer was irradiated. The initially prepared *trans* layer was illuminated with UV light and after the surface pressure reached a stable value associated with the *cis* configuration, the layer was switched back to *trans*-configuration again with visible light. This illumination cycle was repeated at least three times. The irradiation time ranged from 60 to 300 s. The surface pressure was recorded during the entire process.

### X-ray measurements

The X-ray measurements were carried out at the Liquid Interface Scattering Apparatus (LISA) at P08 of PETRA III at DESY^33,34^ and I07 of Diamond^35^. At Diamond, a photon energy of 12.5 keV and Pilatus 100 k with a pixel size of 172 µm × 172 µm was used. A photon energy of 18 keV and a Lambda GaAs 750 k detector with 55 µm × 55 µm pixel size were used at DESY. To clearly separate the switching effects from e.g*.* beam damage the reproducibility of the X-ray measurement is crucially important. For that reason, a beam damage analysis was done at the beginning of each beam time to ensure the reproducibility of the X-ray measurements. To reduce beam damage from oxygen and background scattering the trough was permanently being flushed with helium. Additionally, the X-ray dose was adjusted until repeating measurements gave the same results. To reduce further radiation damage, the trough was moved after each measurement in horizontal direction by 3 mm. With this procedure it was possible to achieve repeatable XRR measurements for a *trans* to *cis* to *trans* illumination cycle of the glycolipid doped DPPC membrane. The XRR data reduction for DESY was carried out using python-based software. The recorded 2D detector images were processed with a region of interest of 2750 µm × 550 µm which results in an angular resolution in *q*_*z*_ direction of 0.14 degree, to extract the specular reflected beam. The background was removed by subtracting the average background intensity at 0.03 degrees next to the used region of interest. For the measurements at Diamond the beamline script was used with an angular resolution of 0.13 degrees in *q*_*z*_ direction for data reduction. The background was corrected at 0.05 degrees and a footprint correction was carried out to compensate for over illumination at low angles.

### X-ray fitting

The X-ray reflectivity fitting was carried out with a modified version of Refnx^36^ allowing direct R/R_Fresnel_ fitting. A two slab model was used to describe the head and chain region of the lipid monolayer. The roughness between the layers, the fronting and backing is described by an error function. Refnx uses the Abeles matrix formalism^37^ to calculate the reflectivity based on a model SLD profile which describes the structure across the interface. In order to obtain physically meaningful results this model is often constrained somehow, for example by limiting the possible SLD based on the physical properties of the sample. For lipids such as DPPC it is common to use the molecular volumes derived from other measurements^38^ or MD simulations. However, even for well studied lipids the volumes are not necessarily applicable when applied to monolayers^39^. For this work data on molecular volumes of the azobenzene glycolipids used is not readily available, and this is further confused by the mixing, the photoswitching and variable surface pressure. Instead, we use a two slab model to describe the head and chain region of the lipid monolayer, with fitted variables of thickness, SLD and roughness for each layer. Due to the relatively high roughness only 2 to 3 times smaller than the layer thickness, micro slicing of 0.5 Å was used^40^ This approach means that, while the variables are correlated, we can use a consistent methodology for all samples. There is inevitably some error in the structural detail for each layer, but the collective parameters, such as the total thickness, are reliable and can be compared as a function of the experimental variables. Errors were calculated using the MCMC sampling tool from Refnx. Further details of the fitting process are given in the supporting information.

## Supplementary Information

Supplementary Information.

## Data Availability

The datasets generated during and/or analysed during the current study are included in the supplementary information files.
