# Supplementary material for: Photoinduced bidirectional switching in lipid membranes containing azobenzene glycolipids
Source: Sci Rep. 2023 Jul 16;13:11480. doi: 10.1038/s41598-023-38336-x (PMC10350456; doi:10.1038/s41598-023-38336-x)
Supplement: Supplementary file 1 — Supplementary Information. [file 41598_2023_38336_MOESM1_ESM.docx]

Supplementary Information
Photoinduced bidirectional switching in lipid membranes containing azobenzene glycolipids

Jonas E. Warias,^a^ Franziska Reise,^b^ Svenja C. Hövelmann,^a,d,e^ Rajendra P. Giri,^a,e^ Michael Röhrl,^b^ Jule Kuhn,^a^ Malte Jacobsen,^a^ Kuntal Chatterjee,^a†^ Thomas Arnold,^c††^ Chen Shen,^d^ Sven Festersen,^a^ Andrea Sartori,^a†††^ Philipp Jordt,^a^ Olaf M. Magnussen,^a,e^ Thisbe K. Lindhorst,^b^ Bridget M. Murphy^a,e*^

^a^Institute of Experimental and Applied Physics, Kiel University, Leibnizstr. 19, D-24118 Kiel, Germany
^b^Otto Diels Institute of Organic Chemistry, Kiel University, Otto-Hahn-Platz 3-4, D-24118 Kiel, Germany ^c^Diamond Light Source, Harwell Science and Innovation Campus, Didcot, OX11 ODE, UK

^d^Deutsches Elektronen-Synchrotron DESY, Notkestraße 85, D-22607 Hamburg, Germany

^e^Ruprecht Haensel Laboratory, Kiel University, D-24118 Kiel, Germany

Email: murphy@physik.uni-kiel.de

Table of Contents

[UV-VIS spectroscopy 2](#_Toc133850938)

[Switching kinetics 4](#_Toc133850939)

[Switching cycle comparison starting from *trans* or *cis* 5](#_Toc133850940)

[Surface pressure response 1(C12)/DPPC at LC’ 6](#_Toc133850941)

[Pure azobenzene glycolipid isotherms 7](#_Toc133850942)

[XRR Fit values 7](#_Toc133850943)

[DPPC area per molecule fit calculation and comparison 9](#_Toc133850944)

# UV-VIS spectroscopy

Photo irradiation was performed using either a UV light LED (2.7 mW, λ = 365 nm for *trans* to *cis* isomerization), or a visible light LED (2.6 mW, λ = 455 nm for *cis* to *trans*) respectively. For the UV-VIS measurements, the azobenzene derivatives were dissolved in Chloroform (CHCl_3_) to yield a 1 mM solution and kept in the dark for the duration of at least one week, to thermally relax the azobenzene into the *trans* state (*trans* dark). The *trans* dark UV/Vis spectrum was recorded first, keeping the sample in the dark. Immediately following irradiation for 60 seconds, at 365 nm (*trans* to *cis* isomerization) and 455 nm (*cis* to *trans* isomerization) respectively, a UV/VIS spectrum were recorded. All measurements were done at room temperature with a Cary 4000 double beam spectrometer (Varian Inc.). The UV-Vis spectra were collected in the range from 260 to 600 nm with 1 nm resolution. Detailed information regarding the setup used was published previously by our group^1^.


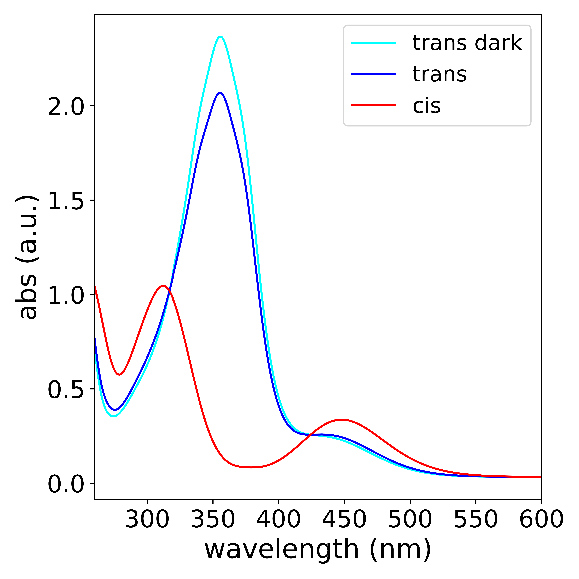


Figure S1: UV/Vis spectrum of **2**(C16) in Chloroform (1 mM) at room temperature in the thermally relaxed (*trans* dark), *trans* and *cis* state. The data is in accordance with previously published data.^2^

In Figure S1, a spectrum is shown for a 1 mM C16 solution in chloroform at room temperature as discussed previously.^2^ The absorption spectra switching from the *trans* to *cis* state show an increase in absorbance for the n-π* transition, a simultaneous decrease for the π-π* transition indicating the formation of the respective *cis* isomer. Data from our previous paper^2^ has been further analyzed to give a complete frame of reverence. In contrast to the thermally completely switched *trans* configuration, a photostationary state consisting of a mixture of *trans* and *cis* isomers rather than an all-*cis* or all-*trans* configuration is observed after photoswitching. This derives from the overlap in the UV-VIS absorption of the *trans* and *cis* isomers. For the UV-induced *trans*-*cis* isomerization a high absorption contrast at 365 nm is observed. Assuming the *cis* absorption to be negligible, the remaining measured absorption has to result from the remaining *trans* state molecules. By comparing the absorption with the fully thermally switched *trans* state it can be estimated, that over 96% of the molecules are in *cis* configuration after switching. A low absorption of the molecules in the *cis* state at this wavelength would imply an even higher population of the *cis* fraction. In comparison, the lower absorption contrast at 455 nm results in a decreased intensity ratio between the *cis* and the thermally relaxed *trans* state spectra indicating 82 % of the molecules are within the *trans* state.

# Switching kinetics

To calculate the switching time of the Langmuir monolayer structure the mean lifetime $\tau$ was determined as $\Delta\pi=A(1-e^{{-t}/\tau})$ with time $t$, change in surface pressure $\Delta\pi$ and amplitude $A$.


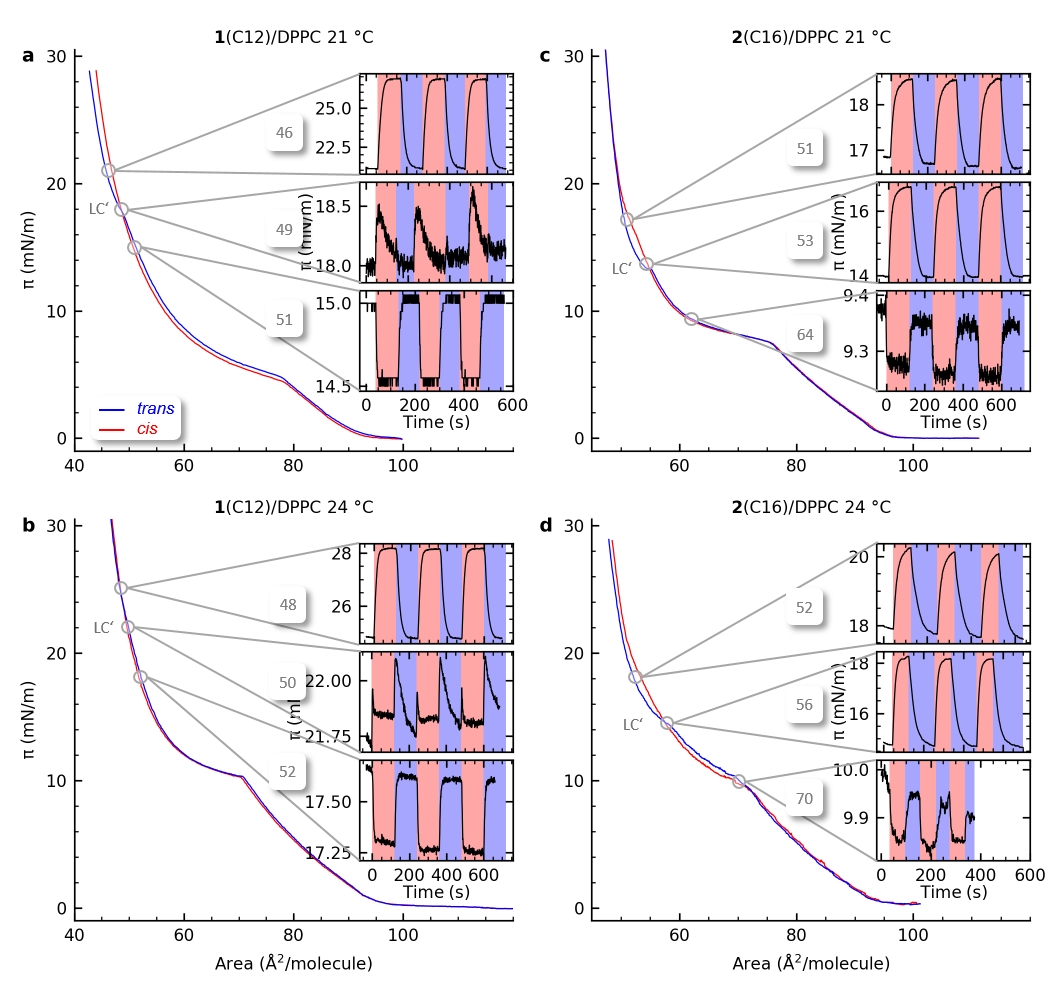


Figure S2: Isotherm for the mixed glycolipid **1**(C12)/DPPC monolayer are shown in a), b) and for **2**(C16)/DPP monolayer in c), d) at 21 °C and 24 °C for *cis*- (red) and *trans* (blue) conformation. Insets show the surface pressure response to photoswitching of embedded azobenzene glycolipids at selected APMs below, close to and above LC’. The switching from *trans* to *cis* and back was induced by irradiation with UV (365 nm) depicted with red background and visible light (455 nm), depicted with blue background. Data are shown on the same scaling for ease of comparison. Switching was always started from the *trans* isotherm.

# Switching cycle comparison starting from *trans* or *cis*

Figure S3 shows the surface pressure change upon photoswitching for **2**(C16)/DPPC at 21°C around LC’. First a *trans*-isotherm was compressed up to 14 mN/m and some switching cycles starting with UV-light where taken at constant APM value (blue line). Second a *cis*-isotherm was compressed up to the highest surface pressure value from the first *trans* started switching cycle. Again a switching cycle starting with visible light where taken (red line).


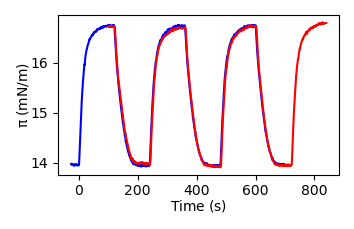


Figure S3: Surface pressure change upon photoswitching for **2**(C16)/DPPC at 21°C starting from the *trans*-isotherm (blue) compared to the start from the *cis*-isotherm (red).

# Surface pressure response 1(C12)/DPPC at LC’

Figure S4 shows the surface pressure response (Δπ) of **1**(C12)/DPPC at 21°C to irradiation with UV and visible light, as shown in Figure 3a of the main paper or Figure S2a. The transient increase in pressure leveling off to the starting pressure can be explained by a coexistence of the two azobenzene glycolipid **1**(C12) conformations as postulated in Figure 5 with their different switching behavior. This conformational coexistence was simulated for the *cis* to *trans* (blue) switching with a quantity ratio of 12 % to 88 % (Figure S4). Despite deviations, the curve can be presented sufficiently. Nevertheless, other effects cannot be excluded. The cyan (above LC’) and green (below LC’) dotted lines describe the individual fit components with the proposed quantity ratio. Regarding the *trans* to *cis* switching (red) a simulation only including the quantity ratio is not possible due to the shift of switching times above and below LC’. Nevertheless, coexistence of the two states is likely for the *trans* to *cis* switching, even though a structural reorientation of the azobenzene glycolipid is dominant.


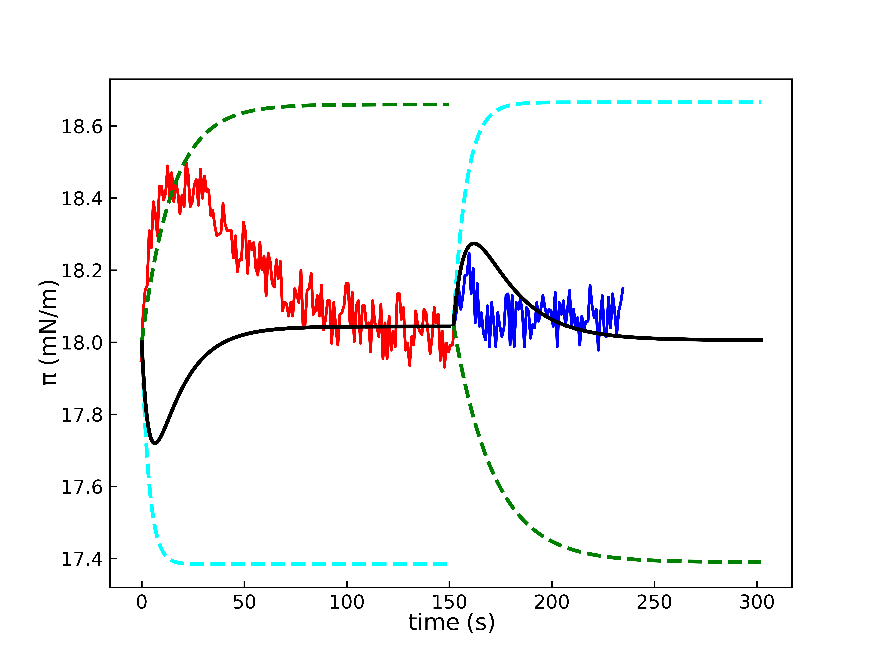


Figure S4: Surface pressure response (Δπ) to photoswitching of **1**(C12)/DPPC at constant APM around LC’ at 49 Å²/molecule. The switching from *trans* to *cis* (red) and back to the *trans* state (blue) were induced by irradiation with UV (365 nm, 1.07 mW/cm^2^) and visible light (435 nm, 1.15 mW/cm^2^) respectively. Black lines indicate a simulated quantity ratio of 12:88 percent for a mixture of azobenzene glycolipid **1**(C12) states above (cyan) and below (green) LC’ within the DPPC matrix.

# Pure azobenzene glycolipid isotherms


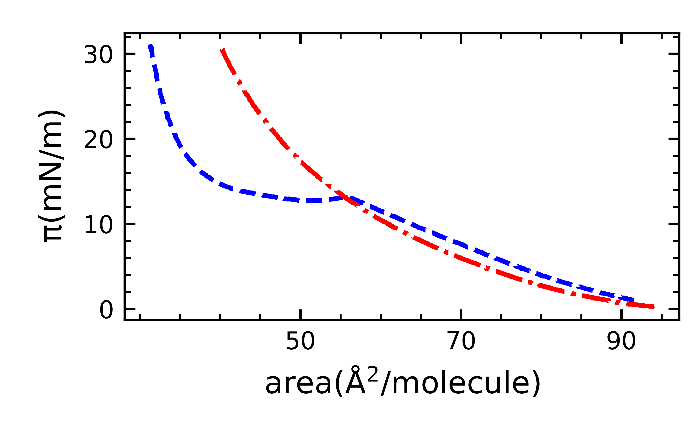

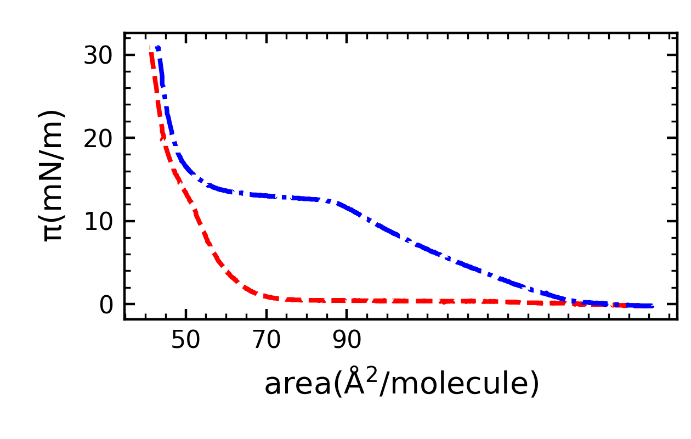


Figure S5: π-isotherms of azobenzene glycolipid **1**(C12) (left) and azobenzene glycolipid **2**(C16) (right) monolayers in *trans* (blue) and *cis* (red) conformation at 21°C.

# XRR Fit values

To distinguish the impact of the photoswitching from other effects and reduce the number of free parameter in the fit, we used constraints during the fitting process for parameters that did not change significantly over the fitting process. This was done in particular for the head thickness and roughness as these were strongly correlated. We also constrained the head thickness and roughness at different surface pressures for samples with similar single fit values. The same method was used for the DPPC monolayer as a reference to determine the influence of the fitting method on the physical parameter.

Table S1: Fit parameters for the DPPC at 21°C, DPPC at 24°C, **1**(C12)/DPPC and **2**(C16)/DPPC monolayers obtained from fits of the X-Ray reflectivity data via a two-slab model. Shown are the layer thickness *l*, the scattering length density *SLD* and the roughness *σ* values between the different parts of the layering system (water, head and tail). The *SLD_water_* was fixed to the measured value of 9.47±0.05·10^-6^ Å^-2^.

| *π*  mN/m | Area from isotherm  Å²/molecule | *l_tail_*  Å | *l_head_*  Å | *l_total_*  Å | *SLD_tail_*  10^-6^ Å^-2^ | *SLD_head_*  10^-6^ Å^-2^ | *σ_tail_*  Å | *σ_head_*  Å | *σ_water_*  Å |
| --- | --- | --- | --- | --- | --- | --- | --- | --- | --- |
| **DPPC** 24°C | | | | | | | | | |
| 10 | 60 | 9.6±0.2 | 7.9±0.5 | 17.5±0.7 | 8.5±0.1 | 12.1±0.2 | 3.1±0.1 | 3.1±0.2 | 4.0±0.2 |
| 15 | 52 | 14.7±0.2 | 8.6±0.2 | 23.3±0.4 | 9.3±0.1 | 13.2±0.2 | 3.1±0.1 | 5.4±0.2 | 3.9±0.2 |
| 18 | 50 | 15.5±0.3 | 8.6±0.3 | 24.1±0.6 | 9.5±0.1 | 13.1±0.2 | 3.1±0.1 | 5.4±0.3 | 3.2±0.2 |
| 30 | 46 | 16.4±0.3 | 8.6±0.3 | 25.0±0.6 | 9.5±0.1 | 13.3±0.2 | 3.4±0.1 | 5.4±0.3 | 3.3±0.2 |
| **DPPC** 21°C | | | | | | | | | |
| 10 | 51 | 14.5±0.6 | 7.8±0.5 | 22.3±1.1 | 8.1±1.0 | 13.0±1.2 | 3.5±0.5 | 6.0±0.9 | 5.0±0.6 |
| 15 | 49 | 15.5±0.3 | 8.1±0.4 | 23.6±0.7 | 9.0±0.8 | 13.0±0.5 | 3.5±0.1 | 5.0±0.3 | 4.1±0.2 |
| 18 | 48 | 16.0±0.3 | 8.1±0.4 | 24.1±0.7 | 9.1±0.8 | 13.0±0.5 | 3.3±0.1 | 5.0±0.3 | 3.6±0.2 |
| 30 | 46 | 16.6±0.3 | 8.1±0.4 | 24.7±0.7 | 9.2±0.8 | 13.0±0.5 | 3.5±0.1 | 5.0±0.3 | 3.9±0.2 |
| **1**(C12)**/DPP**C | | | | | | | | | |
| 15 *trans* | 51 | 14.1±0.2 | 9.2±0.3 | 23.3±0.5 | 9.3±0.1 | 12.5±0.2 | 3.6±0.1 | 5.0±0.1 | 4.4±0.1 |
| 14.3 *cis* | 51 | 13.4±0.1 | 9.2±0.1 | 22.6±0.2 | 9.1±0.1 | 12.0±0.2 | 3.6±0.1 | 5.0±0.1 | 4.5±0.1 |
| 18 *trans* | 49 | 15.0±0.1 | 9.2±0.1 | 24.2±0.2 | 9.4±0.1 | 12.2±0.2 | 3.6±0.1 | 5.6±0.1 | 3.7±0.1 |
| 18 *cis* | 49 | 15.0±0.1 | 9.2±0.1 | 24.2±0.2 | 9.2±0.1 | 12.9±0.2 | 3.6±0.1 | 5.6±0.1 | 4.1±0.1 |
| 20 *trans* | 46 | 15.8±0.1 | 9.2±0.1 | 25.0±0.2 | 9.2±0.1 | 12.8±0.2 | 3.5±0.1 | 4.4±0.1 | 3.0±0.1 |
| 26.5 *cis* | 46 | 16.0±0.1 | 9.2±0.1 | 25.2±0.2 | 9.0±0.1 | 12.5±0.2 | 3.5±0.1 | 4.4±0.1 | 3.0±0.1 |
| **2**(C16)**/DPPC** | | | | | | | | | |
| 10 *trans* | 70 | 10.0±0.2 | 7.0±0.5 | 17.0±0.7 | 8.5±0.2 | 12.7±0.3 | 3.3±0.1 | 3.0±0.2 | 6.5±0.5 |
| 9.9 *cis* | 70 | 10.0±0.2 | 7.0±0.4 | 17.0±0.6 | 8.2±0.1 | 12.3±0.2 | 3.3±0.1 | 3.0±0.1 | 6.1±0.5 |
| 15 *trans* | 56 | 10.0±0.1 | 8.8±0.3 | 18.8±0.4 | 9.0±0.1 | 12.2±0.2 | 3.6±0.1 | 3.5±0.2 | 9.0±0.7 |
| 18.4 *cis* | 56 | 13.3±0.3 | 8.8±0.3 | 22.1±0.6 | 8.5±0.2 | 12.7±0.3 | 3.3±0.1 | 5.2±0.3 | 5.5±0.4 |
| 18 *trans* | 52 | 12.7±0.2 | 8.8±0.3 | 21.5±0.5 | 8.1±0.2 | 13.3±0.3 | 3.3±0.1 | 5.2±0.3 | 7.0±0.6 |
| 20.3 *cis* | 52 | 14.2±0.2 | 8.8±0.3 | 23.0±0.5 | 8.5±0.1 | 13.5±0.3 | 3.3±0.1 | 5.2±0.2 | 5.8±0.3 |

# DPPC area per molecule fit calculation and comparison

For pure materials it is possible to calculate the area per molecule from the fitted parameters extracted from XRR. This is done for DPPC (Table S2) using ${SL}/{(SLD l)}$, with the theoretical scattering length *SL* of 6897·10^-6^ Å, the scattering length density SLD of the tail SLD_tail_ and the tail layer thickness l_tail_ . For higher surface pressures from 15 mN/m a small but almost constant offset of up to 2 Å²/molecule is observed. The observed error is within the 5 % error of the isotherm surface pressure and can be neglected. At 10 mN/m the larger offset of 8 Å²/molecule at 21 °C or even 24 Å²/molecule at 24 °C can be explained by several phenomena. On the one hand, the fitting procedure has influence here. On the other hand, the measurement was done at the beginning of the LE/LE-LC phase transition. The known micro domain formation may influence the measurement, if the domain size is above the coherence length. In an attempt to find a good solution the following approaches were tested. First, an incoherent fit of the measured XRR curve is not feasible as it would require too many free parameter and there are not enough features in the XRR shown. Second, to obtain a fit with a reasonable area value, other fit parameters returned unphysical results (e.g. unrealistically small SLD values for the head group). Another attempt following Schalke^3^ to ignore the high *q_z_* regime did not improve the results. We therefore choose to follow a consistent fitting approach as for all other fits since we could achieve physically reasonable values with reasonable errors using the box model in combination with micro slicing, as the shown fit in Figure 4. It describes the measured curve well and enables to describe general trends from the photoinduced switching.

Table S2: Calculated and measured area per molecule for pure DPPC.

| Π  mN/m | area_fit_  Å²/molecule 21°C | area_isotherm_  Å²/molecule 21°C | area_fit_  Å²/molecule 24°C | area_isotherm_  Å²/molecule 24°C |
| --- | --- | --- | --- | --- |
| 10 | 59 | 51 | 84 | 60 |
| 15 | 49 | 49 | 51 | 52 |
| 18 | 47 | 48 | 47 | 50 |
| 30 | 45 | 46 | 44 | 46 |

(1) Krekiehn, N. R.; Müller, M.; Jung, U.; Ulrich, S.; Herges, R.; Magnussen, O. M. UV/Vis Spectroscopy Studies of the Photoisomerization Kinetics in Self-Assembled Azobenzene-Containing Adlayers. *Langmuir* **2015**. https://doi.org/10.1021/acs.langmuir.5b01645.

(2) Reise, F.; Warias, J. E.; Chatterjee, K.; Krekiehn, N. R.; Magnussen, O.; Murphy, B. M.; Lindhorst, T. K. Photoswitchable Glycolipid Mimetics: Synthesis and Photochromic Properties of Glycoazobenzene Amphiphiles. *Chem. - A Eur. J.* **2018**. https://doi.org/10.1002/chem.201803112.

(3) Schalke, M.; Lösche, M. Structural Models of Lipid Surface Monolayers from X-Ray and Neutron Reflectivity Measurements. *Adv. Colloid Interface Sci.* **2000**. https://doi.org/10.1016/S0001-8686(00)00047-6.
